# Fusion of Environmental Sensors for Occupancy Detection in a Real Construction Site

**DOI:** 10.3390/s23239596

**Published:** 2023-12-04

**Authors:** Athina Tsanousa, Chrysoula Moschou, Evangelos Bektsis, Stefanos Vrochidis, Ioannis Kompatsiaris

**Affiliations:** Information Technologies Institute, Center for Research and Technology Hellas, 6th km Charilaou-Thermi Road, 57001 Thessaloniki, Greece; moschouc@iti.gr (C.M.); evanbekt@iti.gr (E.B.); stefanos@iti.gr (S.V.); ikom@iti.gr (I.K.)

**Keywords:** occupancy detection, environmental sensing, sensor fusion, smart buildings

## Abstract

Internet-of-Things systems are increasingly being installed in buildings to transform them into smart ones and to assist in the transition to a greener future. A common feature of smart buildings, whether commercial or residential, is environmental sensing that provides information about temperature, dust, and the general air quality of indoor spaces, assisting in achieving energy efficiency. Environmental sensors though, especially when combined, can also be used to detect occupancy in a space and to increase security and safety. The most popular methods for the combination of environmental sensor measurements are concatenation and neural networks that can conduct fusion in different levels. This work presents an evaluation of the performance of multiple late fusion methods in detecting occupancy from environmental sensors installed in a building during its construction and provides a comparison of the late fusion approaches with early fusion followed by ensemble classifiers. A novel weighted fusion method, suitable for imbalanced samples, is also tested. The data collected from the environmental sensors are provided as a public dataset.

## 1. Introduction

Monitoring occupancy in buildings has been gaining increasing attention and is considered an important feature of smart buildings. Occupancy estimation can be achieved via heterogeneous sensors and is quite useful for multiple purposes, such as managing energy efficiency in buildings by automatically controlling light and heating [1]; enhancing safety by detecting a presence in a room that should have been vacant or even monitoring the availability of positions in parking lots. Occupancy-driven building systems can monitor a variety of parameters affecting energy consumption. In [2], the authors proposed WinLight, a novel lighting control system that can adjust the intensity of lighting according to the occupancy status of a room.

The current work focuses on occupancy detection via environmental sensors installed in a building under construction. Occupancy detection can be achieved via multiple heterogeneous sensors, such as ultrasonic ones that require motion for detection but are not that accurate, and PIR sensors which also detect motion and have similar disadvantages as ultrasonic sensors; however, they are more energy saving [3]. Visual sensors have been widely used for occupancy detection, achieving high levels of accuracy; however, they are considered quite intrusive and, in some cases, cannot be installed at all due to privacy issues. Thus, visual sensors are mostly found in applications regarding parking lots [4].

Environmental sensors are installed in buildings to provide information about the air or temperature condition, but when combined, they can also be useful in detecting occupancy, in a non-intrusive way. Their low-cost and non-intrusive nature, has increased the interest towards environmental sensors; however, they are reported to not provide such accurate results. Among the available environmental sensors, the carbon dioxide concentration is found to be highly correlated with the number of occupants; thus, it is extensively used in relevant tasks. Temperature, humidity, and air pressure are also suitable for occupancy detection and are often used in combination with sensor fusion frameworks [3].

### 1.1. State of the Art

A combination of sensors to achieve improved accuracy in occupancy detection can be achieved with data fusion methods or deep learning approaches. A summary of deep and transfer learning applications for detection can be found in this review [5]. Transfer learning is usually adopted to overcome some common challenges of occupancy data, such as missing labels. According to the review, the majority of works employ Convolutional Neural Networks and Multilayer Perceptron Algorithms. Cameras, microphones, and environmental sensors were used in [6] for indoor occupancy detection. The authors trained individual models for each data modality and combined them using an ensemble approach. More specifically, variations in spatiotemporal pattern network models were used to perform fusion at the feature level and the decision level. Transfer Kernel learning is used in an occupancy detection scheme in [7] after performing feature extraction and selection on time series data.

Environmental sensors are also used in [8], where the authors propose the fusion of environmental sensors and WiFi signals for occupancy detection. An Artificial Neural Network, Support Vector Machines (SVM), and k-Nearest Neighbor (kNN) are used for predictions separately on environmental and wifi data, as well as on the fused dataset. The fusion was performed at an early level, with concatenation of the two datasets. In order to avoid intrusive sensors, the authors of [9] used environmental sensors combined with activity detection models to increase their performance. The human activity detection models translated the raw environmental data (temperature) into general activities like the door handle-touch event and the water-usage event.

Hybrid sensing frameworks combine indoor environmental sensors with multiple other sources, such as outdoor environmental conditions and energy consumption information. Such a hybrid sensing system is proposed in [10], where the authors performed occupancy prediction using indoor information about air quality, WiFi-connected devices, and energy consumption combined with outdoor weather information. The authors also proposed a novel feature selection method incorporated in deep learning architectures. Indoor environmental sensors were combined with WiFi connection information in [11]. The authors proposed an adaptive lasso feature selection method combined with neural networks to perform occupancy detection.

Sensors for temperature, humidity, light, and ${\mathrm{CO}}_{2}$ were used in [12] to detect if a space was occupied. The authors assessed the performance of four classifiers on multiple combinations of the available sensors and information about the weekday and time of recording. The same sensors were used in [1], where a sparse auto encoder was used for feature learning and then three classifiers were applied on the feature set for occupancy detection, in order to monitor the building’s energy consumption. The same sensors were used in [13], where an occupancy detection system was used based on sparse auto encoders, to learn features from the data, combined with classifiers such as Softmax, SVM, and Liblinear. The authors used a public dataset with recordings from the respective sensors and labels extracted from time-stamped pictures. Unfortunately, this way of labeling is not always possible in real environments due to potential violation of privacy issues.

A combination of sensors often leverages the individual performance and achieves higher accuracy in the prediction. A novel fusion method for the combination of cameras and thermal sensors is proposed in [14]. The authors processed the outputs of the two heterogeneous sensors in a distributing computing platform and the outputs are combined via IoT (Internet-of-Things) in a master controller. Feature-level sensor fusion was used in [15]. The authors installed several environmental sensors in an indoor residential building and extracted features and selected subsets using feature selection methods based on correlations. The features were then concatenated and used as inputs in several classifiers.

### 1.2. Contribution of This Paper

The concatenation of measurements of different sensors is one of the most standard methods of early fusion and is usually selected for real-applications. Late fusion methods may be more sophisticated and, in many cases, perform better than concatenation; however, they are more time consuming. In this work, an evaluation of the performance of multiple late fusion methods is presented, followed by a comparative study with early fusion applications. One of the main contributions of the paper is that the methodologies are evaluated on real data collected from an actual construction site under real conditions. Thus, one of the main challenges is the imbalance between the two classes. For this reason, the performance of a novel weighted late fusion technique is tested. The technique is based on detection rates of classifiers and was proposed by the authors of this paper in [16]. The collection of data used to perform the experiments of this paper is made public in order to contribute to the limited selection of relevant open data for occupancy detection.

The dataset comprises of data from five environmental sensors, namely sound level, temperature, relative humidity, dust, and air pressure, with measurements produced every 15 min. The data were collected from a real construction site, a building that will host offices. The sensors were installed on different rooms of each floor. Camera images are not available in order not to violate privacy rules. Similar environmental sensors can be found in [17]; however, that dataset is suitable for occupant counting. Another public dataset for occupancy detection is ROBOD [18] with different variables than the current dataset, such as Wi-Fi-connected devices, energy consumption information, and outdoor weather conditions.

The contribution of this work can be summarized in the following:

- The performance evaluation of multiple fusion methods on environmental sensors for occupancy detection, including a novel weighted fusion method based on detection rates;
- The comparison of late and early fusion, as well as the evaluation of the performance of individual environmental sensors;
- A publicly available dataset with recordings from a real demo site, from five environmental sensors and a variable measuring activity, that can be used as ground truth.

The rest of the paper is organized as follows: Section 2 explains the experimental setups and gives the theoretical background of the methods applied; Section 3 demonstrates the results of the application; and the final section concludes the findings of the paper.

## 2. Materials and Methods

This section provides an overview of the general methodology and the theoretical background of the methods used. The classification problem considers m=5 sensors and k=2 classes; however, the following methodology can be applied even with a higher number of classes. The environmental sensors used were sound level, temperature, relative humidity, dust, and air pressure.

**Figure 1:**
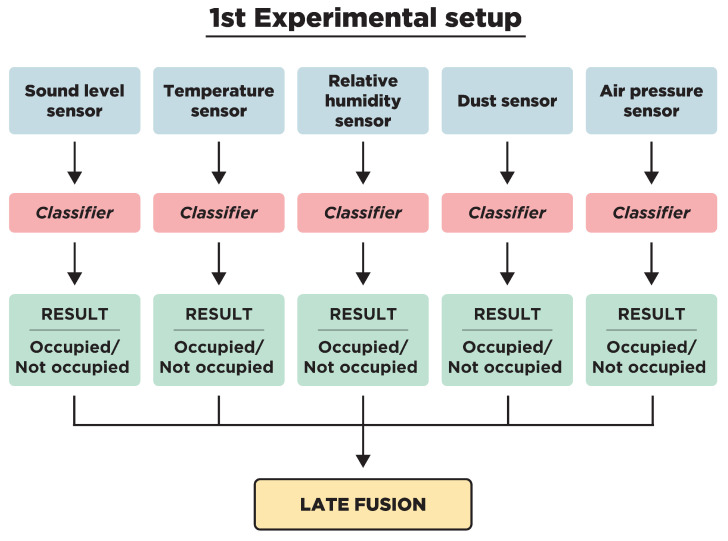
Flowchart of the first experimental setup.

**Figure 2:**
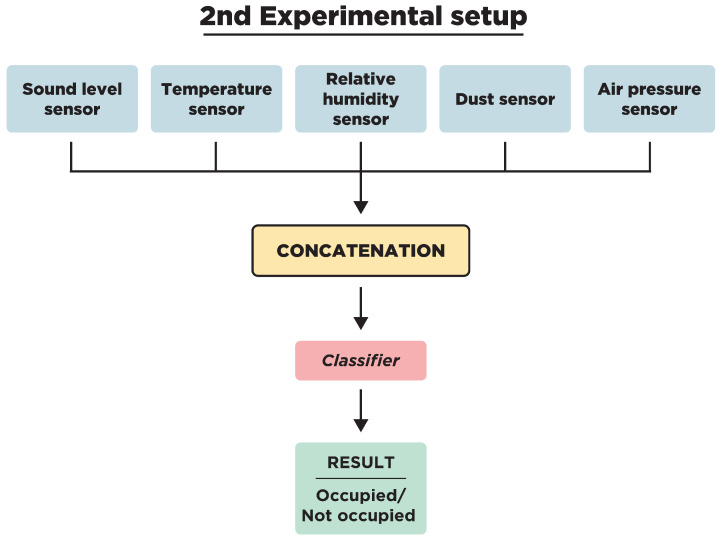
Flowchart of the second experimental setup.

Two different experimental setups were designed to compare the performances of late fusion methods with the early fusion of all available sensors (Figure 1 and Figure 2). In the first experimental setup (Figure 1), several classifiers were applied to each individual sensor to assess their performance in detecting the presence of occupants in a room. Following this, the results of these classifiers were combined using various late fusion methods. Although certain environmental sensors can detect occupancy individually, the main purpose of training classifiers separately was to test whether the combination of individual results encompasses more information than the classifiers trained on concatenated sensor measurements. Since the data were imbalanced, weighted methods, such as the weighted late fusion framework and the class-based weighted late fusion framework proposed in [16], were applied. The second experimental setup (Figure 2) consists of the early fusion of the available sensor readings per room via concatenation. Two ensemble classifiers were applied to the concatenated sensor readings for occupancy detection.

### 2.1. First Experimental Setup

The following classifiers were used in the first experimental setup for occupancy detection based on each of the environmental sensors: SVM, a popular supervised learning algorithm that finds an optimal hyperplane to separate different classes [19]; Random Forest (RF), an ensemble learning method that combines multiple decision trees to make predictions. It improves accuracy and reduces overfitting [20]; kNN, a non-parametric classification algorithm that assigns a class label to an unknown sample based on the majority vote of its k-nearest neighbors [21]; Gradient Boosting (GB), an ensemble learning method that combines multiple weak prediction models, typically decision trees, to create a strong predictive model [22]; Gaussian Naive Bayes (GNB), a probabilistic classifier that applies Bayes’ theorem with the assumption of independence between features, assuming that the features follow a Gaussian distribution [23]; and Decision Tree (DT), a tree-based classifier that partitions the feature space based on a set of if–else conditions, leading to a hierarchy of decision rules [24].

The classification pipeline consists of a training and a testing stage. During the training stage of a model, the classifier is trained on an individual sensor. Proceeding with the testing stage, the trained model outputs for each test case (a) a predicted label and (b) a probability score P(x), expressing how possible it is for each test case to belong to a class. So, after training a specific type of base learner for every sensor, a probability vector (1) is formed in the following way:

(1) $$P_{ij}=\left\{p_{ij}\left(x_{i1}\right),\ldots ,p_{ij}\left(x_{in}\right)\right\}$$

where i=1,…,m, j=1,…,k and $x_{i}=\left[x_{i1},\ldots ,x_{in}\right],i=1,\ldots ,m$ are the input data from the sensor *i*. Practically, (1) expresses the probability of the output of a base learner trained on sensor *i* to be classified as *j*. In order to utilize the information provided via the *m* sensors, the probability vectors of the test set are combined in different ways, according to the late fusion technique that is being used each time.

The basic late fusion methods that were applied are averaging and major voting [25], weighted average [26], and stacking [27]. In the **averaging** method, the average of the predictions of the test set from all the sensors for each basic classifier is computed as in (2):

(2) $$P_{average,j}=\frac{\sum_{i=1}^{m}P_{ij}}{m},$$

and then the class with the highest probability is assigned to the input.

In the **major voting method**, the predicted labels of the test set from all the sensors for each basic classifier are considered as a “vote”. For each data point of the test set, the prediction with the majority of the votes is used as the final prediction. This method can only be applied if the number of the voters, in this specific case, the number of the sensors, is odd.

The **weighted average method** is an extension of the averaging method, only in this case, all sensors are assigned different weights, defining the importance of each sensor for the final prediction. As a result, (2) is modified in the following way (3):

(3) $$P_{w.average,j}=\sum_{i=1}^{m}w_{i}P_{ij},$$

The weight value $w_{i}$ that corresponds to a sensor is a one-dimensional real number and via (3) is multiplied with the probability score of every class (1).

The weights have to satisfy the expression in (4):

(4) $$\sum_{i=1}^{m}w_{i}=1,$$

and can be assigned either via a trial and error process or by carefully examining the effect each sensor has on the final result. A modification of the method is proposed in this work, by replacing the hand-picking weights with an optimization method. The optimizer computes the optimal set of weights for the sensors by applying the weighted average method using the predicted probabilities of the train set and trying to maximize a desired metric. The desired metric is computed via the final score (3). The set of weights that produces the optimal results is then used with the predicted probabilities from all the sensors in the test set to make the final prediction. In this paper, the optimization technique that was chosen was the Bayesian optimization [28] and it was implemented with the python library bayes_opt.

The **stacking** method is a more advanced late fusion method. In this case, the predicted probabilities of the train set from all the sensors and a specific type of base model are concatenated together to form a new training set. With the new training set, a new model is built and then used to make predictions on the test set.

The **weighted late fusion** framework is similar to the weighted average method, but in this case, each sensor is assigned *k* weight values equal to the number of the classes. A new weight vector (5) is formed:

(5) $$W_{ij}=\left\{w_{i1},\ldots ,w_{ik}\right\},$$

where i=1,…,m, j=1,…,k and $w_{i}j$ corresponds to sensor *i* and class *j*, respectively, and is a one-dimensional real number. Combining (3) and (5), a final score for each class is formed. The final predicted label for each test case is the class with the maximum final score (6).

(6) $$Score_{j}\left(x\right)=\sum_{i=1}^{m}W_{ij}P_{ij},$$

Through (6), the *k* weight values are multiplied with the probability score of the corresponding class (1).

For the following research, two different approaches were followed for the computation of the weights. In the first approach, referred to as weighted late fusion 1, the weight values that are used for each sensor are based on the sensors’ ability to detect the true predictions for each class. This is expressed via the detection rate (DR) of each class [16] that focuses on the true positives and not all true finding of an algorithm. For multiclass problems, the comparison of the predicted and actual classes is performed with the one vs. all approach, meaning that the class to be evaluated consists the “positive” findings and all the rest the “negative” findings. The detection rate is computed during the training stage of each sensor. The detection rate (7) is equal to the ratio of the true positives for a class (TP) to all the predicted values, including the true negatives (TN), the false positives (FP), and the false negatives (FN).

(7) $$DR=\frac{TP}{TP+TN+FP+FN}$$

To assist the recognition of classes not so easily detected, the weights are set equal to the supplementary of the detection rate (8).

(8) $$W_{ij}=1-DR_{ij},$$

For the second approach, named weighted late fusion 2, the weight values were computed via an optimization method and were not based on the detection rate. The optimization method aims to find the best set of weights that maximize a desired metric on the train set. The desired metric is computed via the final score (6). The set of weights that produces the optimal results is then used with predicted probabilities from all the sensors in the test set, as in (6), to make the final prediction. The optimization technique that was chosen was the Bayesian optimization.

The implementation of the **class-based weighted late fusion** framework is similar to the weighted late fusion, but in this case, the weights and the predicted probabilities are combined in a modified way. Instead of the sum of the product of the weight values and the predicted probabilities from all the sensors, the sum of the following expression (9) was used to create the final score (10):

(9) $$AP_{ij}=\alpha W_{ij}+\left(1-\alpha \right)P_{ij},$$

(10) $$Score_{j}\left(x\right)=\sum_{i=1}^{m}AP_{ij},$$

The adaptation parameter α is assigned values ranging from 0 to 1. For our experiments, two cases were examined: (a) using a fixed adaptation parameter for all the sensors, and (b) using an optimized adaptation parameter for each sensor, computed with an optimization technique. The optimization method aims to find the best adaptation parameter α that maximizes a desired metric on the train set. The desired metric is computed via the final score (10). The adaptation parameter that produces the optimal results is then used with predicted probabilities from all the sensors in the test set, as in (10), to make the final prediction. The optimization technique that was chosen was the Bayesian optimization.

### 2.2. Second Experimental Setup

For the second experimental setup, two of the most effective classifiers were tested, Light Gradient Boosting Machine (LGBM) and Extremely Randomized Trees (ExtraTrees). They were used to detect occupancy from concatenated sensor readings. LGBM is a gradient boosting framework that uses tree-based learning algorithms. It is designed to be distributed and efficient with the following advantages: faster training speed and higher efficiency, lower memory usage, better accuracy, support for parallel and GPU learning, and capable of handling large-scale data [29]. Its unique selling point is its attention to leaf-wise (best-first) tree growth, in contrast to the level-wise tree growth found in other tree-based algorithms. By choosing the leaf with maximum delta loss for growth, it tends to achieve better results for the same number of leaves. ExtraTrees is an ensemble learning method fundamentally similar to Random Forests. However, it differs in the way it splits nodes. Instead of searching for the most discriminative thresholds, like RF does, thresholds are drawn randomly for each candidate feature and the best of these randomly-generated thresholds is picked as the splitting rule. This randomness tends to increase the model’s bias but also its robustness, which can result in better models. Its high computational efficiency and performance in multi-class problems make it a powerful classifier for sensor fusion [30].

### 2.3. Dataset Description

The dataset used in this paper can be found online (Released on December 2023, http://doi.org/10.5281/zenodo.8203278). It consists of environmental sensor measurements from the construction of a 27-story office building, with a final height of 110 m. Five sensors measuring sound level, temperature, relative humidity, dust, and air pressure were installed on specific locations on different floors. The data were collected from February 2021 to May 2022. Measurements were produced every 15 min. A numeric variable that detected any activity in a room was used as the ground truth of whether the room was occupied. In the current paper and in the dataset made public, the five rooms with the most records (Room A, Room B, Room C, Room D, and Room E) were selected to demonstrate the results of the experiments.

Prior to initiating model training, the data were meticulously cleaned to account for any missing values and outliers. This step was crucial in ensuring a reliable model training process. The data originating from each room were divided into training and test sets, adhering to a 70:30 ratio. Initially, the activity level, serving as the model’s label, was a continuous value. However, it was decided that setting a threshold at 0.70 would transform the problem into a binary classification task, making it possible to predict whether a room is occupied or not.

## 3. Results

Two different experimental setups were designed to compare the performances of late fusion methods with the early fusion of all available sensors. In the first experimental setup, classifiers were applied to each individual sensor to assess their performance in detecting the presence of occupants in a room. The six most well-performing ones are reported here. Following this, the results of these classifiers were combined using various late fusion methods. The combined sensor reading methods utilized early fusion by concatenating the available sensors and afterwards applying two ensemble classifiers for occupancy detection. The performances of the two experimental setups were compared and the results revealed a higher performance of the ensemble classifiers applied on the concatenated sensor readings.

In order to assess the performance of the classifiers and compare the obtained results, the overall accuracy was not an appropriate criterion, due to the unbalanced nature of the data, so the overall balanced accuracy (11) of each algorithm is reported for all the configurations. In most of the rooms, the activity labels respond to 12–14% of the sample, with the exception of Room D, where there are 41.8% activity labels.

Balanced Accuracy (11) is the average of Sensitivity (12) and Specificity (13). Sensitivity is also known as the true positive rate or recall and it measures the proportion of true positives out of all positive predictions that were made via the model. Specificity is also known as the true negative rate and it measures the proportion of correctly identified negatives over the total negative predictions that were made via the model.

(11) $$Balanced\;Accuracy=\frac{Sensitivity+Specificity}{2},$$

(12) $$Sensitivity=\frac{TP}{TP+FN},$$

(13) $$Specificity=\frac{TN}{TN+FP},$$

### 3.1. First Experimental Setup

The following described process was applied for each room. The number of the sensor is m=5, and since the label is Boolean, the classification problem consists of k=2 categories (Occupied, Not Occupied). For the recognition of occupancy based on the information from each sensor individually, several classifiers were tested, with the results of the six that performed better reported here, namely SVM, kNN, RF, GB, GNB, and DT. For all of the classifiers, the hyperparameters were set to the default ones that are used in the open source machine learning library *Scikit-learn*.

**Figure 3:**
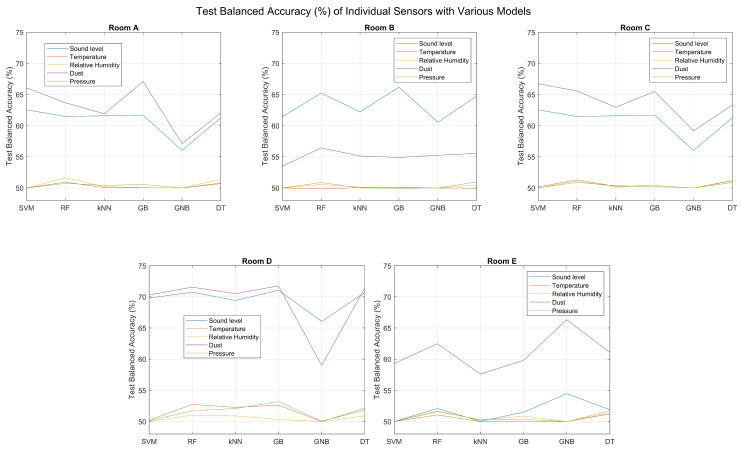
Test Balanced Accuracy (%) of Individual Sensors with Various Models for Room A, Room B, Room C, Room D, and Room E.

After training all types of base learners on each individual sensor, the trained models were used on the test set and the results for the balanced accuracy are shown in Figure 3. From the results, it is clear that the classification based on each individual sensor does not perform well. The best performing sensors are the Dust and Sound level ones, since in every room they achieve the highest balanced accuracy values compared to the rest. The difference is evident for all the rooms and fluctuates between 7% and 20%. These values appear in Room E and Room D, respectively. These results were expected, as Room D has the most balanced data, while Room E has the most unbalanced data. Regarding the performance of classifiers, the GB and SVM demonstrate superior performance, with the exception of Room E, where the GNB achieves the highest balanced accuracy. However, the differences with the other classifiers are mostly insignificant in the majority of cases. In general, the results suggest that the balanced accuracy is more influenced by the sensor type and data quality rather than the choice of classifier.

Following the application of various classifiers on individual sensors, the results of all available sensors per room were combined, using multiple late fusion techniques. Initially, the performance of standard late fusion methods was tested, such as averaging, major voting, weighted average, and stacking. The general guidelines for each method is described in Section 2.

For the **weighted average method**, the weights can be assigned by carefully examining the effect each sensor has on the final result or by using an optimization method to produce these weights. The criterion for the optimization methods is the balanced accuracy. For this implementation, three cases were considered: (a) Hand-picked weights [0.4, 0, 0.1, 0.4, 0.1] (Weighted Average 1), (b) Hand-picked weights [0.5, 0, 0, 0.5, 0] (Weighted Average 2), and (c) Bayesian-optimized weights (Weighted Average 3). The previously mentioned weights correspond to this sequence of sensors: Sound level, Temperature, Relative humidity, Dust, and Pressure. The hand-picked weights were chosen after carefully examining the results of each subplot of Figure 3, where in the majority of the results, the Sound level and the Dust sensors gave the best results, with a significant difference from the rest. This justifies the selection of hand-picked weights in the second set where only these two sensors were given a value and the other three were not considered at all in the final decision. The Relative Humidity and Pressure sensors are the next best in terms of balanced accuracy; thus, they were given an increased weight value in the first set of hand-picked weights. The same set of values were applied to all the rooms. However, the weights could be different for each room and adjusted each time according to the performance of each sensor.

For the **stacking method**, the predicted probabilities of the train set for all the sensors are taken into consideration to build a new model. This model is used for making predictions on the test set. To implement the stacking method, three different algorithms were used as the new model that has to be trained: (a) AdaBoosting, (b) Bagging, and (c) XtraTree. AdaBoosting, short for Adaptive Boosting, is a powerful ensemble learning method that combines multiple weak classifiers to create a strong classifier [31]. Bagging, short for Bootstrap Aggregating, is another ensemble learning technique that combines the predictions of multiple independent classifiers trained on different subsets of the training data [32].

The results from the implementation of the above-mentioned fusion methods for all the rooms are presented in Table 1, Table 2, Table 3, Table 4 and Table 5. At the end of each table, the best score for the balanced accuracy from the use of individual sensors is reported as well as the maximum increase that the fusion methods achieve, compared to the best performing individual sensor. Averaging and Major voting give far worse results than the best performance from the individual sensors. The best late fusion methods seem to be the more advanced ones, which are AdaBoosting, Bagging, and XtraTree.

In the weighted average method, the optimization seems to improve the results for the base learners SVM, kNN, and GB, but not for the RF, GNB, and DT. The problem is that during the optimization process, the weights seem to overfit the training data (train accuracy for these models ∼99%). Trying to randomly split the train set during each optimization epoch did not seem to improve the results.

The results revealed that the above-mentioned late fusion methods do not improve the performance for the base models RF and DT. For all the rooms with unbalanced data, the best score from a late fusion method is worse than the best score from the individual sensors. For Room D, although there is improvement for all base learners, the RF and DT are the ones with the lowest increase in the balanced accuracy.

In the following, the implementation of more advanced weighted fusion methods is described. As it is explained in Section 2 of this paper, for the weighted late fusion framework, each sensor is assigned multiple weight values (5), not just one, equal to the number of the classes. This is why in our case, two weights correspond to each sensor. Since the two classes are imbalanced, these values are based on the detection rate of each class. The detection rate is computed during the training stage of each sensor. The detection rate of the “No Occupancy” class (DR_0) (14) is equal to the ratio of the true negatives (TN) to all the predicted values, and the detection rate of the “Occupancy” class (DR_1) (15) is equal to the ratio of the true positives (TP) to all the predicted values.

(14) $$DR_{0}=\frac{TN}{TP+TN+FP+FN},$$

(15) $$DR_{1}=\frac{TP}{TP+TN+FP+FN},$$

To assist the recognition of classes not so easily detected, the weights were set as equal to the supplementary of each detection rate (16), (17), as proposed in [16].

(16) $$W_{0}=1-DR_{0},$$

(17) $$W_{1}=1-DR_{1},$$

Another approach for the computation of the weights, instead of relying on the detection rate, is to use an optimization method. The algorithm used for the optimization is the Bayesian optimization. The results from these two frameworks are presented with the results of the next section. The weighted late fusion method with the detection rate-based weights will be referenced as Weighted Late Fusion 1, and the one with the optimized weights as Weighted Late Fusion 2.

For the class-based weighted late fusion framework, the adaptation parameter α is assigned values ranging from 0 to 1. For the present experiments, two cases were examined (a) using a fixed adaptation parameter α = 0.25 (Class-based Late Fusion 1), and (b) using an optimized adaptation parameter α for each sensor (Class-based Late Fusion 2). The algorithm used for the optimization is the Bayesian optimization. The results from weighted late fusion frameworks and from the class-based weighted late fusion framework for all the rooms are given in Table 6, Table 7, Table 8, Table 9 and Table 10. At the end of each table, the best score for the balanced accuracy from the use of individual sensors is reported as well as the maximum increase that the weighted late fusion frameworks achieve.

The implementation of the weighted late fusion frameworks achieved the best results for the occupancy detection in a room, compared to the previously mentioned fusion methods. For all the base learners, the balanced accuracy increases and, for some cases, it reaches values higher than 80%. Specifically, these results were achieved with the use of GB classifiers as basic models for the late fusion. The only exception to the increase in the balanced accuracy occurs in Room D, which is the one with the most balanced data. The implementation of the weighted late fusion framework using RF and DT classifiers as base learners seems to have negative results towards the balanced accuracy.

### 3.2. Second Experimental Setup

These predictions were based on concatenated sensor readings from the five sensors available in each room, a method that diverges from the first experimental setup where classifiers were positioned on each sensor, followed by subsequent fusion. This approach was also adopted during the Ashvin project, serving as a foundation for occupancy predictions.

The unique characteristics of the chosen models, which are both ensemble methods, negated the need for data scaling. A combination of grid search and cross validation was leveraged to refine the model’s hyperparameters, thereby ensuring an optimal training outcome. The models’ performance was assessed using balanced test accuracy, a metric that compensates for any imbalances in class distribution. Upon evaluation, as illustrated in Table 11, it was discovered that the XtraTree model held an advantage over the LGBM model in predicting room occupancy, with an average increase in accuracy ranging between 1% and 2% per room.

In summary, the analysis indicates a certain superiority of the XtraTree algorithm over the LGBM algorithm in the context of predicting room occupancy, utilizing various sensor inputs. In comparison to the results of the first experimental setup, although the increase for the balanced accuracy for the basic late fusion methods is significant compared to the individual sensor classification, it does not exceed the performance of XtraTree and LGBM for the combined sensor readings. The only exception to this is for Room D (which is the one with the most balanced data), where with the use of GNB as the base model and XtraTree as the fusion method, the balanced accuracy reaches a value of 85.94%, whereas the values for combined sensor readings with XtraTree and LGBM are 82.9% and 81.8%, respectively.

The results are similar when it comes to the more advanced late fusion frameworks, where the balanced accuracy for the weighted late fusion and the class-based weighted late fusion does not exceed the performance of XtraTree and LGBM for the combined sensor readings. The only exception to this is for Room A, where with the use of GB as base models and the Weighted late fusion 2 as the fusion method, the balanced accuracy reaches a value of 81.27%, whereas the values for combined sensor readings with XtraTree and LGBM are 80.3% and 79.2%, respectively.

## 4. Discussion

In this paper, the performance of fusion methods in detecting occupancy from environmental sensors was presented. The experiments were conducted on data collected from an office building during its construction. Usually, frameworks that need to make predictions in real time use concatenated vectors of data which are fed to a classifier and produce results in less time than late fusion frameworks. Late fusion methods, however, may achieve higher accuracy, especially when individual base classifiers perform well. For research purposes, the current paper evaluates the performance of several late fusion methods in comparison with ensemble classifiers applied on concatenated data. Furthermore, three variations in weighted late fusion frameworks were proposed, namely (a) weights based on the detection rate, (b) weighted averaging using Bayesian optimization, and (c) weighted late fusion with optimized weights calculated again from Bayesian optimization.

Different types of late fusion methods were selected in order to explore which ones could perform well, given the nature of the data collected in a real demo site. Weighted fusion methods can enhance the performance of the best of the individual classifiers and decrease the effect of poor performing ones. The recently introduced weighted fusion method that utilizes detection rates assists in the prediction of classes not easily recognized. Although a wide variety of late fusion methods was explored, the ensemble classifiers applied on concatenated sensor data achieved the highest performance scores.

The collection of data derived from the environmental sensors is provided as a public dataset. It is important to note that the data were collected under real conditions, in an actual construction site. The labeling of the data, regarding the presence of people in a room, was not performed via cameras, but a sensor detecting activity.

## 5. Conclusions

The extensive experiments of this paper revealed that the two ensemble models applied on the concatenated sensor readings outperformed the late fusion methods used to combine the results of individual sensors. In the context of occupancy detection, and based on this research, LGBM and ExtraTrees have shown to be particularly efficient. The former is effective in capturing complex nonlinear relationships between features, while the latter provides robustness against noise and outliers commonly found in sensor data. This allows for a more accurate and stable prediction of the occupancy state, outperforming the other tested classifiers.

## Figures and Tables

**Table 1:** Test Balanced Accuracy (%) for fusion methods for Room A.

	SVM	RF	kNN	GB	GNB	DT
Averaging	50.00	50.96	50.10	50.00	50.10	51.67
Major Voting	50.00	52.16	50.19	50.24	50.00	51.88
Weighted Average 1	54.46	56.02	54.81	55.62	53.05	56.18
Weighted Average 2	55.17	60.28	58.43	60.02	57.53	60.29
Weighted Average 3	61.58	51.00	63.81	67.09	57.53	50.78
AdaBoosting	65.51	52.67	64.29	68.71	71.11	51.97
Bagging	73.44	51.77	64.19	68.20	77.05	51.21
XtraTree	72.05	52.24	63.60	69.12	76.21	50.81
Best Individual Sensor score	66.09	63.69	61.92	67.09	57.13	62.07
Max Increase	+7.35	−3.41	+2.37	+2.03	+19.92	−1.78

**Table 2:** Test Balanced Accuracy (%) for fusion methods for Room B.

	SVM	RF	kNN	GB	GNB	DT
Averaging	50.00	50.35	50.00	50.00	50.00	51.15
Major Voting	50.00	51.18	50.00	50.05	50.00	50.83
Weighted Average 1	50.73	54.53	52.28	52.50	52.09	54.71
Weighted Average 2	51.02	57.61	55.29	56.18	59.80	56.92
Weighted Average 3	58.61	50.66	62.68	66.18	59.32	50.34
AdaBoosting	64.32	51.99	63.16	67.66	66.78	51.05
Bagging	74.32	50.34	62.27	68.31	77.10	50.46
XtraTree	71.10	51.73	61.91	68.56	76.83	50.95
Best Individual Sensor score	61.46	65.23	62.23	66.18	60.56	64.76
Max Increase	+12.86	−7.62	+0.93	+2.38	+16.54	−7.84

**Table 3:** Test Balanced Accuracy (%) for fusion methods for Room C.

	SVM	RF	kNN	GB	GNB	DT
Averaging	50.00	51.16	50.13	50.00	50.04	52.61
Major Voting	50.00	53.48	50.17	50.35	50.00	52.80
Weighted Average 1	54.35	55.47	55.24	53.53	55.54	55.55
Weighted Average 2	56.05	60.98	59.31	61.08	62.21	60.61
Weighted Average 3	62.20	50.41	64.42	65.50	61.02	52.70
AdaBoosting	70.98	51.52	64.36	68.60	73.56	50.72
Bagging	75.98	51.87	64.70	70.09	84.20	51.25
XtraTree	73.50	51.66	63.53	69.89	82.62	50.91
Best Individual Sensor score	66.75	65.57	62.97	65.50	61.02	63.34
Max Increase	+9.23	−4.59	+1.45	+4.59	+23.18	−2.73

**Table 4:** Test Balanced Accuracy (%) for fusion methods for Room D.

	SVM	RF	kNN	GB	GNB	DT
Averaging	68.65	66.83	71.18	72.40	62.34	63.11
Major Voting	50.07	65.56	59.49	55.76	50.02	64.58
Weighted Average 1	74.51	76.19	76.57	76.98	69.62	75.58
Weighted Average 2	75.78	76.75	76.24	77.27	69.62	76.06
Weighted Average 3	75.65	52.97	74.44	76.52	69.81	58.87
AdaBoosting	77.05	56.62	74.23	74.73	74.94	54.20
Bagging	81.59	58.78	72.22	76.20	85.63	54.47
XtraTree	79.53	54.54	72.43	76.72	85.94	52.02
Best Individual Sensor score	70.30	71.56	70.51	71.77	66.05	71.43
Max Increase	+11.29	+5.19	+6.06	+5.50	+19.89	+4.63

**Table 5:** Test Balanced Accuracy (%) for fusion methods for Room E.

	SVM	RF	kNN	GB	GNB	DT
Averaging	50.00	50.00	50.00	50.00	50.00	50.10
Major Voting	50.00	49.99	50.00	50.00	50.00	49.99
Weighted Average 1	50.00	51.13	49.99	50.46	52.97	52.24
Weighted Average 2	50.00	53.75	50.43	51.35	61.28	58.07
Weighted Average 3	50.00	50.46	49.99	50.46	53.97	50.26
AdaBoosting	65.07	52.81	58.27	64.02	67.04	53.13
Bagging	68.23	53.82	58.29	65.80	78.16	51.77
XtraTree	67.52	52.17	58.27	65.79	77.06	51.89
Best Individual Sensor score	59.30	62.47	57.60	59.81	66.33	61.09
Max Increase	+8.93	−8.65	+0.69	+5.99	+11.83	−3.02

**Table 6:** Test Balanced Accuracy (%) for weighted late fusion for Room A.

	SVM	RF	kNN	GB	GNB	DT
Weighted Late Fusion 1	76.52	69.82	76.47	79.75	75.93	68.33
Weighted Late Fusion 2	78.04	53.84	75.56	81.27	77.54	55.95
Class-based Late Fusion 1	50.00	53.54	51.41	50.81	51.33	54.42
Class-based Late Fusion 2	72.30	63.80	75.81	78.95	67.29	58.40
Best Individual Sensor score	66.09	63.69	61.92	67.09	57.13	62.07
Max Increase	+11.95	+6.13	+14.55	+14.18	+20.41	+6.26

**Table 7:** Test Balanced Accuracy (%) for weighted late fusion for Room B.

	SVM	RF	kNN	GB	GNB	DT
Weighted Late Fusion 1	63.77	70.03	74.90	80.36	74.34	67.93
Weighted Late Fusion 2	63.92	53.40	76.79	81.51	75.95	53.12
Class-based Late Fusion 1	50.00	52.11	50.47	50.14	51.10	53.31
Class-based Late Fusion 2	65.58	61.59	76.42	79.04	65.90	58.73
Best Individual Sensor score	61.46	65.23	62.23	66.18	60.56	64.76
Max Increase	+4.12	+4.80	+14.56	+15.33	+15.39	+3.17

**Table 8:** Test Balanced Accuracy (%) for weighted late fusion for Room C.

	SVM	RF	kNN	GB	GNB	DT
Weighted Late Fusion 1	78.01	72.72	74.68	83.72	77.09	68.57
Weighted Late Fusion 2	80.37	54.16	78.01	83.35	80.34	52.91
Class-based Late Fusion 1	50.13	54.18	52.18	50.75	52.57	54.84
Class-based Late Fusion 2	72.36	65.39	76.41	82.33	75.32	60.20
Best Individual Sensor score	66.75	65.57	62.97	65.50	61.02	63.34
Max Increase	+13.59	+7.15	+15.04	+18.22	+19.32	+5.23

**Table 9:** Test Balanced Accuracy (%) for weighted late fusion for Room D.

	SVM	RF	kNN	GB	GNB	DT
Weighted Late Fusion 1	50.04	68.20	66.55	63.58	52.84	65.11
Weighted Late Fusion 2	75.44	58.11	72.61	76.97	71.43	52.23
Class-based Late Fusion 1	75.77	67.10	74.42	77.51	69.38	63.08
Class-based Late Fusion 2	75.74	67.90	74.33	77.76	69.57	64.30
Best Individual Sensor score	70.30	71.56	70.51	71.77	66.05	71.43
Max Increase	+5.47	−3.36	+3.91	+5.99	+3.52	−6.32

**Table 10:** Test Balanced Accuracy (%) for weighted late fusion for Room E.

	SVM	RF	kNN	GB	GNB	DT
Weighted Late Fusion 1	64.23	74.55	78.38	81.93	81.56	71.65
Weighted Late Fusion 2	53.48	53.39	78.54	80.75	81.52	52.48
Class-based Late Fusion 1	50.00	50.46	50.00	50.00	51.68	53.25
Class-based Late Fusion 2	56.69	57.34	75.97	72.31	69.71	63.78
Best Individual Sensor score	59.30	62.47	57.60	59.81	66.33	61.09
Max Increase	+4.93	+12.08	+20.94	+22.12	+15.23	+10.56

**Table 11:** Test Balance Accuracy (%) for XtraTree and LGBM Models for all rooms.

Room	XtraTree	LGBM
A	80.3	79.2
B	89.6	87.6
C	86.0	86.3
D	82.9	81.8
E	81.7	83.4

## Data Availability

The dataset used for this paper is available in the Zenodo repository. Release date: December 2023. (http://doi.org/10.5281/zenodo.8203278).

## Abbreviations

The following abbreviations are used in this manuscript:

SVM	Support Vector Machines
kNN	k-Nearest Neighbor
IoT	Internet-of-Things
GB	Gradient Boosting
GNB	Gaussian Naive Bayes
DT	Decision Tree
LGBM	Light Gradient Boosting Machine
RF	Random Forests
